# Development of a microchip capillary electrophoresis method for determination of the purity and integrity of mRNA in lipid nanoparticle vaccines

**DOI:** 10.1002/elps.202100272

**Published:** 2021-12-19

**Authors:** Jessica Raffaele, John W. Loughney, Richard R. Rustandi

**Affiliations:** ^1^ Analytical Research & Development, Merck & Co., Inc. West Point PA 19486 USA

**Keywords:** Covid‐19, Lipid nanoparticles, Microchip CE, mRNA vaccine

## Abstract

Messenger RNA (mRNA)‐based vaccines are advantageous because they can be relatively quicker and more cost efficient to manufacture compared to other traditional vaccine products. Lipid nanoparticles have three common purposes: delivery, self‐adjuvanting properties, and mRNA protection. Faster vaccine development requires an efficient and fast assay to monitor mRNA purity and integrity. Microchip CE is known to be a robust technology that is capable of rapid separation. Here, we describe the development and optimization of a purity and integrity assay for mRNA‐based vaccines encapsulated in lipid nanoparticles using commercial microchip‐based separation. The analytical parameters of the optimized assay were assessed and the method is a stability indicating assay.

**Abbreviations**
HMW: high molecular weight
LNPs: lipid nanoparticles
mRNA: messenger RNA

## 1 Introduction

In recent years, nucleic acid based technologies including messenger RNA (mRNA) and plasmid DNA (pDNA) have been investigated extensively for treatment of genetic disorders, cancer prevention and therapy, as well as vaccines [1, 2]. Although there is currently no approved DNA vaccine [3], two mRNA vaccines for the current pandemic coronavirus, SARS‐CoV‐2, were granted approval for emergency human use by the FDA [4]. Compared to more traditional inactivated or live‐attenuated virus vaccines, mRNA vaccines provide several advantages including simpler and faster vaccine manufacturing. These advantages are mainly because mRNA vaccine manufacturing processes do not require growth and maintenance of host cells or live viruses that are associated with other vaccine platforms such as live or inactivated viruses or subunit protein vaccines. The mRNA used in vaccines can be prepared in an *in vitro* cell‐free transcription reaction, thereby reducing potential concerns of cell or virus related impurities. mRNA vaccines are a promising approach to improving the world's response to disease outbreaks [5].

mRNA is known to be less stable than DNA due to its susceptibility to hydrolysis and the abundance of ribonucleases. Although there are reports that naked mRNA vaccines have been shown to be stable for several months at temperatures at or above 4°C [6, 7], protecting mRNA from degradation by encapsulating within lipid nanoparticles (LNPs) has been reported [8, 9]. Besides protecting mRNA from degradation, LNPs are used as a delivery mechanism to enhance antigen expression and induced T‐cell response [10]. Even after the mRNA is encapsulated within LNPs, it is still necessary to monitor its stability. Hence, it is important to determine the purity of mRNA in vaccines at various stages of the vaccine manufacturing process and determine the mRNA integrity that is a measure of intactness of mRNA over time in the final drug product, which requires a stability indicating method. Demonstrating the integrity of mRNA in vaccines is critical to ensure potency of the vaccine product.

One of the key stability‐indicating parameters of mRNA is degradation. mRNA is typically large, approximately 2000 nucleotides (nt), and there are limited analytical tools to measure the intact mRNA and its degradation products with good resolution. Several chromatography techniques have been reported such as size exclusion [11] and ion‐pair reverse phase HPLC [12], however, both suffer from low resolution and long run time. CGE has shown to be a promising separation technique for analysis of mRNA size that provides high sensitivity and separation efficiency with limited sample and reagent requirements [13, 14]. Most recently, development of a new CGE method using standard commercial CE and long, home‐made coated capillaries has been reported by Lu et al. where the authors were reporting a large ∼2000 nt mRNA separation with good resolution [15]. Although this new method has good resolution, the separation time is rather long hence it is not amenable for analyzing the many samples generated to support mRNA vaccine development. To further increase throughput, CGE can be performed using a microchip format that has a shorter run time, acceptable resolution, and allows for increased sample throughput [16].

The advantages of MCE for proteins have been reported previously [17, 18]. Here, we describe the development and optimization of a purity and stability‐indicating (integrity) assay using MCE for a relatively large mRNA vaccine (∼2000 nt). The optimized new method was qualified by assessing standard analytical parameters following ICHQ2 guidelines. Furthermore, this high‐throughput method of microchip CGE can analyze a 96‐well plate in less than 2.5 h, which is enabling to vaccine process development.

## 2 Materials and methods

### 2.1 Reagents

RNA reagent kits (Catalog# CLS960010) and RNA labchips (Catalog# 760435) were obtained from Perkin Elmer (Waltham, MA). Brij® 58 was obtained from Acros Organics (Pittsburgh, PA). Formamide was obtained from Sigma‐Aldrich (St. Louis, MO). High Range RiboRuler RNA Ladder was obtained from Thermo Fisher Scientific (Norristown, PA).

### 2.2 mRNA‐LNP preparations

LNPs containing mRNA were prepared by our Vaccine Process Development colleagues as previously described [19, 20]. mRNA was encapsulated in LNPs using a self‐assembly process in which mRNA is mixed with a solution of lipids dissolved in ethanol [9]. mRNA‐LNP samples contained mRNA, a cationic lipid, cholesterol, 1,2‐distearoyl‐sn‐glycero‐3‐phosphocholine, and poly(ethylene glycol)2000‐dimyristoylglycerol. Empty LNPs were also prepared using the same process but without mRNA.

### 2.3 Optimized MCE sample preparation

The mRNA‐LNP samples were first diluted to 100 μg/mL mRNA in a solution of 10% (w/v) Brij® 58 in formamide, then further diluted in formamide and 5 μL of 10× sample buffer from the RNA reagent kit for a final total sample volume of 50 μL (10 μg/mL mRNA final concentration). The final formamide concentration in the sample was always >80%. All final sample solutions were heated in a 70°C heating block for 10 min, then cooled on ice for at least 5 min. Samples were transferred to a 96‐well plate. The RNA labchip was prepared as described in the RNA Assay Quick Guide provided by Perkin Elmer without any modifications.

### 2.4 Instrument and software

**Figure 1:**
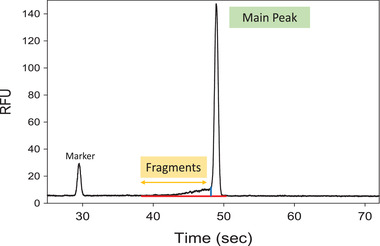
Electropherogram of system suitability sample using final optimized method illustrating the peak integration used to calculate the fragment and main peak areas.

LabChip GXII Touch is an instrument from Perkin Elmer and was used for all experiments. This automated system performs electrophoresis using a “lab on a chip” technology. For the LabChip, gel‐sieving matrix containing a blue fluorescent dye is applied to the separation channel, then sample is electrokinetically injected and mRNA binds to the fluorescent dye. Voltage is applied for separation to occur and mRNA migrates through the sieving gel matrix and separates by size. The mRNA signal is observed by fluorescent detection. Separation time is 70 s for each sample to cover the range of 50–6000 nt of RNA size. The electropherogram for each injection was transferred to Waters Empower 3 chromatography software for analysis. An example electropherogram of mRNA from an mRNA‐LNP sample using the final optimized method is shown in Fig. 1. The red line represents the baseline while the blue line represents a dropped line that separates the main peak from fragments. Each component of main peak, fragments, and high molecular weight (if present) is calculated as a percentage of the total peak area. The mRNA purity or integrity is reported as percent peak area of main peak.

## 3 Results and discussion

### 3.1 Optimization of MCE sample preparation

**Figure 2:**
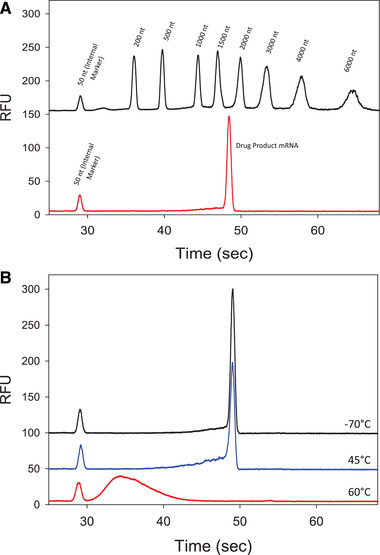
(A) Electropherogram of drug product mRNA (lower red trace) and RiboRuler High Range RNA Ladder containing eight RNA transcripts (upper black trace). (B) Electropherogram comparison of mRNA stored at –70°C (upper black trace) to that stored at 45°C (middle blue trace) and 60°C (bottom red trace) for 4 days. At 60°C, the intact mRNA is reduced into smaller unresolved and broad peak fragments (31–45 s).

The capability of microchip technology of measuring large mRNA from LNPs was first assessed by analyzing an mRNA‐LNP sample that was prepared by dilution in 10% (w/v) Brij® 58 in formamide. Results are shown in Fig. 2A illustrating the mRNA main peak (lower electropherogram red trace) appears close to the expected molecular size of ∼2000 nt and the electropherogram black trace is the RNA ladder standard containing eight RNA transcripts of 200–6000 nt. Furthermore, the instrument was tested to determine if it was capable of measuring mRNA degradation due to heat stress in a sample. Figure 2B illustrates the results of a short initial heat stress experiment that was performed by heating a mRNA‐LNP sample for 4 days at 45°C (blue trace) and 60°C (red trace) in comparison with a control of the same sample stored at –70°C (black trace). The mRNA % main peak area at 49 s decreased after 4 days at 45°C and smaller sized fragments increased as multiple small peaks to the left of the main peak. After 4 days at 60°C, the sample was entirely fragmented with no main peak. The total peak areas of each trace in Fig. 2B are consistent. The group of fragment peaks is very broad and contains many smaller RNA fragments that are not resolved using this method. These data demonstrate that this method is capable of monitoring the stability of heat‐stressed mRNA and detect relatively accurate molecular size. However, further sample preparation optimization needed to be evaluated in order to have a robust assay.

**Figure 3:**
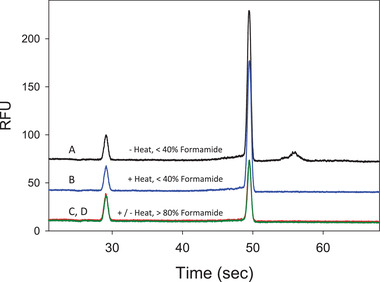
Trace A shows an electropherogram of mRNA diluted in <40% (v/v) formamide but not heated, while trace B shows an electropherogram of mRNA diluted in <40% (v/v) formamide that was heated (70°C for 10 min) during sample preparation. Traces C and D show mRNA treated with >80% (v/v) formamide during sample preparation, with or without heat. Formamide removes the HMW peak regardless of with or without heat treatment.

Several sample preparation parameters including heating conditions, Brij® 58, formamide, and mRNA concentration were evaluated during assay optimization. The detergent Brij® 58 was used to solubilize LNPs and liberate the mRNA. mRNA is known to have tertiary structure and its structure can be denatured in the presence of formamide or heat. The original sample preparation conditions included a final concentration of <40% (v/v) formamide. Figure 3 (trace A) shows that high molecular weight (HMW) size of mRNA (∼4000 nt) at 56 s appears when the formamide concentration is <40% (v/v), while Fig. 3 (trace B) shows that heating at 70°C for 10 min makes the HMW peak disappear while the main peak area increases. This indicates that the HMW peak is likely formed during sample preparation without heat. The short heating time does not cause mRNA degradation since the total peak areas of traces A and B are comparable. On the basis of literature, it is well known that RNA can be denatured in 60% (v/v) formamide and storage of RNA preparations in formamide ensures high RNA stability and protection against ribonucleases [21]. Increasing formamide at the final dilution to maintain a concentration of >80% (v/v) formamide yields a disappearance of the HMW peak with or without heating as shown in Fig. 3 (traces C and D), respectively, indicating that the mRNA was fully denatured in >80% (v/v) formamide. Using this new condition however, the peak intensity of mRNA decreases approximately 50% as compared to the original method. This again shows consistently that upon fully denaturing of mRNA, the fluorescent dye only weakly binds the mRNA as compared to structural mRNA since this dye is considered an intercalating dye.

**Figure 4:**
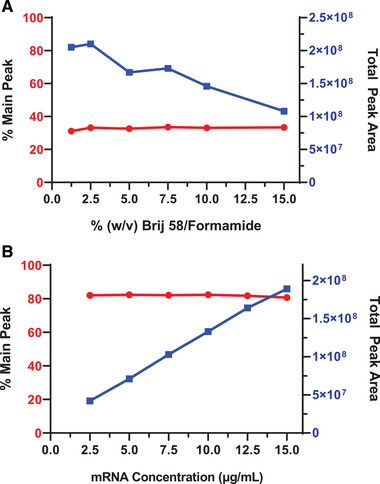
(A) mRNA‐LNP samples were prepared in varying concentrations of Brij® 58 in formamide, from 15 to 1.25% w/v. The total peak area (right *y*‐axis) decreased with increasing % Brij® 58, while the % mRNA main peak remains unchanged at ∼31% (left *y*‐axis). *Note: The sample used in this experiment was a stressed stability timepoint sample; therefore the % mRNA main peak is low*. (B) Linearity study of mRNA‐LNP samples diluted to various concentrations of mRNA. Total peak area (right *y*‐axis) is linear across a range of mRNA concentrations from 2.5 to 15 μg/mL, while the calculated % mRNA main peak (% purity) was also consistent across this mRNA concentration range.

Further optimization was done to evaluate the effect of Brij® 58 concentration on mRNA % purity. Brij® 58 detergent was used to solubilize LNPs and various Brij® 58 concentrations were evaluated to ensure consistent % mRNA purity. The % (w/v) Brij® 58/formamide concentrations of 15, 10, 7.5, 5, 2.5, and 1.25 were evaluated at a target mRNA concentration of 10 μg/mL. Figure 4A displays a plot of % main peak and total peak area with respect to Brij® 58 concentration that shows that while the total peak area decreased with increasing %Brij® 58 concentration, the % main peak stayed relatively the same. It is not fully understood why the total peak area decreased as the Brij® 58 concentration increased. It is possible it is caused by a viscosity effect since Brij® 58 is nonionic hence it should not have any effect from the electrokinetic injection.

### 3.2 Analytical parameters

The assay is intended to measure the % purity and % integrity of mRNA contained in LNPs; therefore, standard analytical parameters were evaluated to understand assay performance before qualification.

*Specificity*: Specificity was assessed by running an empty LNP sample. The empty LNP sample was prepared identically to an mRNA‐LNP sample of ∼1 mg/mL mRNA, as described in Section 2.3. No peaks were observed in empty LNP samples. However, visible RNA peaks were observed in the control RNA ladder and mRNA‐LNP system suitability sample, indicating that LNPs do not contribute to the signal and the signal is only from RNA in the sample.

*Accuracy and precision*: Accuracy was assessed as percent recovery and was evaluated by analyzing a mRNA‐LNP sample of high purity, a mRNA‐LNP sample of low purity, and a 1:1 mixture of the two. The % purity of the 1:1 mixture should theoretically be the average of the % purity of the low purity sample and the high purity sample. Two development samples were used with % purities of 71.7% and 22.9%. The 1:1 mixture of these two samples should produce a % purity of 47.4%. The observed % purity of the 1:1 mixture was 45.0%. The percent recovery was 95% (%recovery = observed % purity/theoretical % purity × 100%). The precision of the new method was obtained by performing intra‐ and interday assay precision measurements (*n* = 3). mRNA‐LNP samples were prepared as described in Section 2.3. The RSD for both repeatability (intra‐assay precision) and intermediate precision (interassay) for the % main peak and % fragments was <1% and <5%, respectively.

*Linearity*: An mRNA‐LNP sample was used to evaluate linearity across a range of mRNA concentrations from 2.5 to 15 μg/mL. The total peak area with respect to this range of mRNA concentrations (2.5–15 μg/mL) is plotted in Fig. 4B and a linear correlation with *R* ^2^ = 0.99 is shown. Within this range, the % main peak was also consistent as shown in Fig. 4B.

*LOQ and LOD*: LOQ and LOD were estimated based on the S/N of measured signals from low mRNA concentration samples compared with the baseline. An mRNA‐LNP sample was diluted across a range of mRNA concentrations from 15 to 0.01 μg/mL. At mRNA concentrations ≤1 μg/mL only a single mRNA peak was detected and fragments were not able to be differentiated from the baseline. For a single RNA peak, the LOD was estimated to be 0.05 μg/mL at an S/N of 3, and the LOQ was estimated to be 0.1 μg/mL at an S/N of 10. The LOQ with respect to % purity was estimated to be 2.5 μg/mL.

Figure 1 shows an electropherogram from an mRNA‐LNP system suitability sample illustrating the internal standard marker, the mRNA fragments, and the mRNA main peak. Table 1 provides analytical data for the % integrity (% main peak) mRNA assay performance using a system suitability sample (*n* = 303) demonstrating % CV <15% for fragments, <5% for main peak, and <20% for total peak area. The system suitability data were acquired by four different analysts, three different LabChip instruments, and at least seven different lots each of labchips and RNA reagent kits.

**Table 1:** System suitability sample

	% Fragments	% Main peak	Total peak area
Mean (*n* = 303)	17.3	82.7	1.27 × 10^8^
% CV	11.2	2.3	14.1

### 3.3 mRNA stability

**Figure 5:**
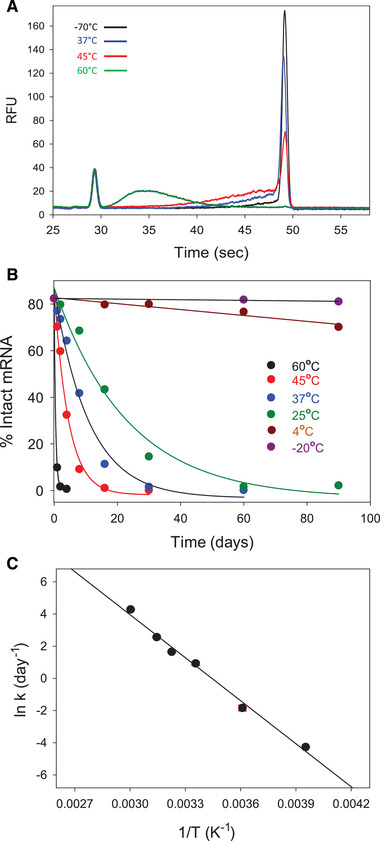
The final optimized method was used to test the mRNA stability of mRNA‐LNP samples. Samples were placed on stability chambers at various temperatures (–20°C, 4°C, 25°C, 37°C, 45°C, and 60°C). (A) An example of electropherograms for four different temperatures after 4 days; (B) % intact mRNA is plotted with days at various temperatures. As the temperature increased, the mRNA integrity (% intact mRNA) decreased. At –20°C and 4°C, the mRNA is relatively stable over 90 days; (C) Arrhenius plot of the natural logarithm of rate constant (*k*) versus 1/*T* indicate a linear relationship.

Naked mRNA is known to be very unstable due to various mechanisms such as heat, metal catalyzed acid‐ or basic based‐hydrolysis, oxidation, as well as the existence of ubiquitous RNase enzyme. Although mRNA fragmentation might follow many pathways, the first‐order kinetic of bond breaking is the major mechanism of its degradation. For example, metal ions catalyzed, alkaline condition, and high temperature activates and accelerates the 2’OH in ribose to attack the neighboring phosphate group to cleave the RNA [22]. It is expected that mRNA‐based vaccine products encapsulated by LNPs would give better stability protection than naked mRNA. Here, we performed a forced degradation study using heat stress at various temperatures (–20°C, 4°C, 25°C, 37°C, 45°C, and 60°C) for up to 90 days. Figure 5A–C illustrates mRNA stability data from heat‐stressed mRNA‐LNP samples at different temperatures. An example set of electropherograms from mRNA‐LNP samples at various temperatures (–70°C black trace, 37°C blue trace, 45°C red trace, and 60°C green trace) for 4 days is shown in Fig. 5A. As the temperature increased, we observed a decrease in % main peak and an increase of % fragments, which resulted in a decrease in % integrity, indicating that the mRNA encapsulated in LNPs was not stable at these higher temperatures after 4 days. The electropherograms of mRNA‐LNP samples stored at –20°C, 4°C, and 25°C are not shown but were similar to that of the –70°C (black trace), indicating that mRNA inside LNPs was stable and minimal changes in the % integrity occurred after 4 days at these temperatures. The starting % purity at time zero –70°C of the mRNA‐LNP product was ∼80% as shown by Fig. 5B. The mRNA integrity decreased with increasing temperatures due to degradation to smaller fragments. The % integrity was 0% after just a few days for the 60°C sample, after ∼20 days for the 45°C sample, ∼30 days for the 37°C sample, and ∼60 days for the 25°C sample. There was no change in mRNA integrity for the –20°C sample over 90 days. The 4°C sample showed a slight ∼10% decrease in mRNA integrity over 90 days, suggesting that in 360 days the product would have decreased 50% from the original starting mRNA. The 90 day stability data suggest that better formulation is needed in order to have long‐term (>2 years) stability of this particular product at temperatures above –20°C. The results demonstrate that the current MCE method can be used to analyze % mRNA purity for release and % mRNA integrity for a stability indicating assay.

Data from two heat stress degradation studies of mRNA‐LNP samples were used to assess the effect of temperature on the reaction kinetics of mRNA degradation. The natural logarithm of the mRNA degradation initial rate constant was plotted against the inverse temperature, as shown in Fig. 5C. The negative slope of this linear plot indicates that the mRNA degradation kinetics follow Arrhenius behavior with activation energy of 74.8 ± 0.8 kJ/mol (*n* = 2). The linear Arrhenius relationship indicates that the reaction kinetic is simple and minimally influenced by structure formation and other factors [22]. The relatively low activation energy correlates with the low melting temperature (33–50°C) measured by DSC in a naked mRNA, and according to Qi and Frishman classification, it falls in the low thermostability (*T_m_* < 46°C) region which has a weaker sequence–structure relationship and less thermostable RNA secondary structure [23]. Furthermore, the activation energy (*E*_a_) of 74.8 kJ/mol calculated from our heat stress experiments is relatively comparable to RNA cleavage in a model system with *E*_a_ of 121 kJ/mol [22] and RNA cleavage by the hammerhead ribozyme with *E*_a_ of 55 kJ/mol [24]. Our data suggest that the degradation pathway for mRNA that has been encapsulated in LNPs requires low activation energy and therefore is susceptible to degradation at relatively low temperatures.

## 4 Concluding remarks

With recent advances in mRNA‐based vaccines, it is essential to have a method that is capable of monitoring the purity and integrity of mRNA. We have developed a quick and reproducible microchip CE method for evaluating the % purity and % integrity of mRNA in mRNA‐LNP vaccine samples. The method utilizes the LabChip GXII Touch instrument from Perkin Elmer, which allows for high‐throughput sample analysis due to the short run time of ∼ 70 s per sample and can run a 96‐well plate in approximately 2.5 h. These advantages are especially useful for vaccine development and the method required minimal changes for optimization of its use for additional mRNA vaccine products. Assay development was optimized mostly with respect to sample preparation since the instrument and separation kit are fixed from Perkin Elmer. Sample treatment was optimized by the addition of high formamide concentration (>80% v/v) and heating at 70°C for 10 min to ensure mRNA was fully denatured. Standard analytical parameters were assessed including specificity, linearity, accuracy, precision, LOD, and LOQ. The method was successfully used to monitor the stability of mRNA in LNPs at various temperatures and is currently used for process and formulation development.

*We gratefully thank Amy Gallagher for analytical support. We also would like to thank our colleagues in Vaccine Process Development, who provided us with all materials*.

*The authors have declared no conflict of interest*.

## Data Availability

Data is proprietary, and it can be available on reasonable request.
